# Prostate Cancer Liquid Biopsy Biomarkers’ Clinical Utility in Diagnosis and Prognosis

**DOI:** 10.3390/cancers13133373

**Published:** 2021-07-05

**Authors:** Milena Matuszczak, Jack A. Schalken, Maciej Salagierski

**Affiliations:** 1 Department of Urology, Collegium Medicum, University of Zielona Góra, 65-046 Zielona Góra, Poland; matuszczakmilena@gmail.com; 2 Department of Urology, Radboud University Medical Centre, 6525 GA Nijmegen, The Netherlands; J.Schalken@uro.umcn.nl

**Keywords:** cancer biomarkers, prostate cancer, liquid biopsy, prognosis, diagnosis, early detection

## Abstract

**Simple Summary:**

In prostate cancer, overdiagnosis and overtreatment is a common problem for clinicians. Accurate diagnosis and prognosis are essential to avoid unnecessary biopsy and to increases the effectiveness of treatment. A new, easy-to-use and non-invasive test based on liquid biopsy biomarkers such as Progensa PCA3, MyProstateScore, ExoDx, SelectMDx, PHI, 4K, Stockholm3 and ConfirmMDx have been developed to improve diagnosis, prognosis and to help guide the decision-making process. This article provides an overview of the above-mentioned commercial tests. The performance and financial aspects of the tests have been compared using available studies. Then the application of biomarker tests as an adjunct to multiparametric MRI in the diagnosis, prognosis and monitoring of prostate cancer has been discussed.

**Abstract:**

Prostate cancer (PCa) is the most common cancer in men worldwide. The current gold standard for diagnosing PCa relies on a transrectal ultrasound-guided systematic core needle biopsy indicated after detection changes in a digital rectal examination (DRE) and elevated prostate-specific antigen (PSA) level in the blood serum. PSA is a marker produced by prostate cells, not just cancer cells. Therefore, an elevated PSA level may be associated with other symptoms such as benign prostatic hyperplasia or inflammation of the prostate gland. Due to this marker’s low specificity, a common problem is overdiagnosis, which leads to unnecessary biopsies and overtreatment. This is associated with various treatment complications (such as bleeding or infection) and generates unnecessary costs. Therefore, there is no doubt that the improvement of the current procedure by applying effective, sensitive and specific markers is an urgent need. Several non-invasive, cost-effective, high-accuracy liquid biopsy diagnostic biomarkers such as Progensa PCA3, MyProstateScore ExoDx, SelectMDx, PHI, 4K, Stockholm3 and ConfirmMDx have been developed in recent years. This article compares current knowledge about them and their potential application in clinical practice.

## 1. Introduction: Prostate Cancer Diagnosis

Prostate cancer (PCa) is the most common cancer in men and the second most common cause of mortality in this population in the United States, with 191,930 new cases and 33,330 deaths in 2020 [1]. Globally, there are approximately 1,276,106 new cases and 358,989 deaths each year [2]. The lifetime risk of being diagnosed with prostate cancer is estimated to be 1 in 9 men, while the risk of death is, fortunately, not as high at around 2% [1].

There is an emerging role for liquid biopsy in PCa, which has excellent potential in preoperative medicine. It is a minimally invasive procedure, analysing even small numbers of targets, which allows its usefulness in screening, diagnosis, prognosis, follow-up and therapeutic management [3]. This review compares the diagnostic and prognostic utility of prostate cancer tests. Good clinical outcomes can be achieved by accurate diagnosis followed by acute treatment or active surveillance in patients with disease located within the gland. There is an unmet clinical need for non-invasive, easily performed diagnostic tests to assess whether a prostate biopsy is indicated. The EAU 2020 guidelines [4] recommend mpMRI before the first biopsy in men with a clinical suspicion of prostate cancer (PCa). Indeed, when mpMRI shows lesions suspicious for PCa (i.e., PI-RADS ≥ 3), targeted biopsy (TBx) and systemic biopsy (SBx) are recommended in patients who have not had a previous biopsy. It therefore represents an important diagnostic tool, and its combination with biomarkers further improves the accuracy of the initial diagnosis of PCa.

**Figure 1:**
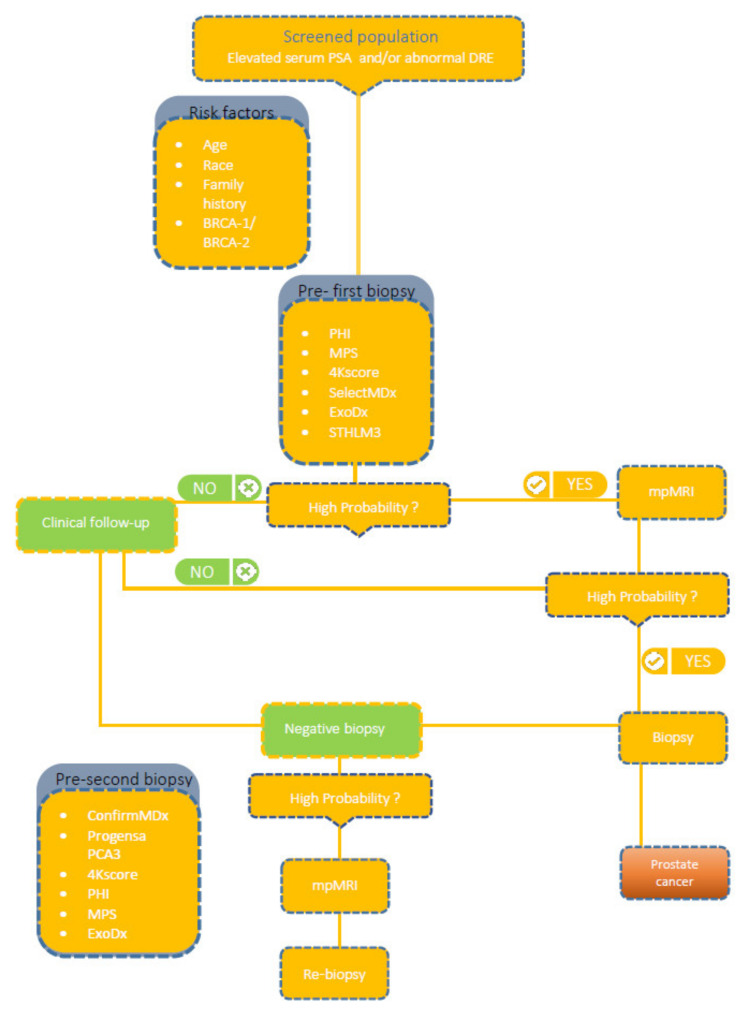
Suggested workflow for utilisation of prostate cancer biomarkers.

The traditional diagnosis of PCa (Figure 1) is based on the assessment of serum prostate-specific antigen (PSA) levels, digital rectal examination (DRE), followed by biopsy under the guidance of transrectal ultrasonography (TRUS). In screening programmes, high PSA levels, despite a normal DRE, lead to the diagnosis of PCa in more than 60% of asymptomatic patients. Serum PSA levels are commonly used for detection, risk stratification and monitoring of PCa [5]; unfortunately, it results in a high number of unnecessary biopsies and detection of asymptomatic cancers with low clinical risk [6]. The reason may be that PSA has a low positive predictive value (~30%) and poor specificity, being organ rather than cancer-specific. This highlights the need to develop more precise methods to identify clinically relevant PCa, such as liquid biopsy-derived biomarkers.

As prostate cancer is a heterogeneous disease, urologists, after identifying the presence of disease during the baseline assessment, focus primarily on assessing the risk group. Risk groups have been classified since 2014 using a classification system with five distinct Grade Groups based on modified Gleason score groups. Group 1 = Gleason score ≤ 6, Group 2 = Gleason score 3 + 4 = 7, Group 3 = Gleason score 4 + 3 = 7, Group 4 = Gleason score 4 + 4 = 8, Group 5 = Gleason score 9 and 10.

Currently, the gold-standard test to confirm all of the above clinical situations is the histopathological result of a prostate biopsy.

Unfortunately, this invasive procedure is painful, expensive and may pose a risk of complications (e.g., infection or sepsis). Furthermore, the procedure is prone to significant sampling error. It is therefore important to avoid unnecessary biopsies [7,8].

Liquid biopsy biomarkers are proving to be a promising new diagnostic and prognostic approach to help optimise the pre-biopsy decision and stratify whether the patient requires treatment or can be monitored under active surveillance.

## 2. Material and Methods

A literature review was performed by searching MEDLINE/PubMed, Google Scholar and and CrossRef electronic databases to identify articles published from January 2000 to October 2020 whose methods included commercially available prognostic and diagnostic prostate cancer liquid biopsy biomarkers or contain information about the characteristics of a relevant biomarker. The search terms included ConfirmMDx, ExoDx, MiPS, PCA3, PHI, SelectMDx, Stockholm3, 4Kscore and and prostate cancer liquid biopsy using search terms database = specific—medical subject headings terms in various combinations appropriate to the research objective. Articles on biomarkers not available in clinical practice or studies based on less than 40 patients were excluded.

## 3. Urine Biomarkers

Urine is obtained non-invasively and contains fluid excreted from the prostate gland, which may contain products from prostate cancer cells [9]. For many urinary biomarkers, performing a DRE is crucial as it increases the excretion of fluid from the prostate. To date, four tests are available with proven clinical utility.

### 3.1. Prostate Cancer Antigen 3 (PCA3)

The Progensa PCA3 test (Hologic Inc., Marlborough, MA, USA) is a test that measures PSA messenger RNA (mRNA) and PCA3 mRNA detectable in the first catch urine sample after DRE. 

Prostate cancer antigen 3 (PCA3, previously called “DD3”) is a long, non-coding RNA (lncRNA) that is overexpressed in 95% of prostate cancers [9]. The test is based on the fact that 60–100 times more PCA3 gene mRNA is detected in prostate cancer cells compared to non-cancerous prostate tissue. 

The PCA3 score is calculated using the Progensa PCA3 method. The test result represents the PCA3/mRNA PSA ×1000 ratio [10].

It is the first urine biomarker test to be approved in 2006 by the European Union, Canada [11] and in 2012 by the FDA. The FDA recommends its use in men ≥ 50 years old to support repeat biopsy decision-making in whom one or more previous prostate biopsies have been negative and for whom repeat biopsy is recommended based on current standards of care [12]. However, some clinical studies [13,14] report the benefits of using the test as early as the first biopsy.

Although the FDA recommends a cut-off PCA3 score = 25, many studies [12,13] suggest a cut-off score of 35 as a more optimal cut-off point. Establishing a cut-off point appears to be of vital importance.

A study [12] evaluated different PCA3 score cut-off points: 10 and 35. For these values, the sensitivity was 87% and 58%, respectively, and the specificity was 28% and 72%, respectively. The results showed that a PCA3 score cut-off of 35 could provide an optimal balance between sensitivity (58%) and specificity (72%) for the diagnosis of PCa and was superior to PSA (Table 1).

Although the study [12] demonstrated high sensitivity and specificity, the ability to improve prostate cancer detection was not shown. For this reason, Wei et al. conducted a prospective validation trial on 859 men [14] to assess whether the PCA3 score could improve the PPV for initial biopsy and NPV for repeat biopsy. The results were PPV = 80% for detecting any PCa at initial biopsy and NPV = 88% at repeat biopsy. This showed that at initial biopsy, a PCA3 score > 60 increases the likelihood of detecting PCa, and at repeat biopsy, a PCA3 score < 20 indicates a low risk of detecting PCa at biopsy [14].

A systematic review and meta-analysis of studies (with a threshold of 35) [13] yielded the following overall values: AUC = 0.734, sensitivity 69% and specificity 65%. These results support the greater clinical utility of cut-off point = 35 than 25 (FDA approved).

Determining the best cut-off value is controversial, especially for primary biopsy—the available studies are very heterogeneous. Several have highlighted that PCA3 does not perform well at a single threshold, showing a high NPV below the low cut-off and a high PPV above the high cut-off, with a grey zone in between—reflecting prostate cancer specificity [14].

Roobol et al., in a publication [15], highlight men with a PCA3 score ≥ 100 and no PCa in a biopsy. This study combines data from the initial and re-biopsies that provided a PPV of 52.2% in men with PCA3 ≥ 100, resulting in almost 50% unexplained high results. To date, there is no explanation why PCA3 scores can be excessively high despite the absence of biopsy-detectable PCa.

Publications [16,17,18] do not show a relationship between PCA3 value and prostate cancer aggressiveness (Gleason score). A high PCA3 level, due to its low specificity, does not help assess prognostic parameters and is therefore of low utility in clinical practice, as it does not provide an answer to how to proceed with the patient. For this reason, to detect patients who require rapid and radical treatment, it is reasonable to use newer, more sensitive and specific diagnostic tools, e.g., SelectMDx (MDxHealth, Inc., Irvine, CA, USA).

Numerous studies [14,19] have shown that the diagnostic value of the test increases when adding other predictors (i.e., age, PSA value, DRE result or prostate volume). Therefore, the producer recommends its use in combination with standard diagnostic parameters [20].

To determine the clinical utility of the PCA3 test in African Americans, Feibus et al. conducted a study [21] (Table 2) on a racially diverse group of men, where 60% of the participants were African American. They demonstrated that the PCA3 test in African Americans also improves the ability to predict the presence of any prostate cancer and high malignancy.

Ochiai et al. [22] (Table 2) examined the diagnostic utility of PCA 3 in Japanese men undergoing prostate biopsy. They achieved a similar diagnostic value to that obtained in men in Europe and the USA. The PCA3 score for men with prostate cancer was significantly higher than for men with negative biopsy results. Furthermore, they showed that also in Japanese men, PCA3 was significantly better than PSA in predicting PCa.

The reported clinical utility of the study mentioned above on the Japanese population and the desire to verify the promising reports of Shen et al. [23] (Table 2) on a small group of Chinese men (prostate cancer patient group (*n* = 35), BPH patient group (*n* = 64)), inspired other researchers to study the Chinese population. Wang et al. conducted a study on a cohort of 500 Chinese men [24] (Table 2). This study showed a moderate improvement in diagnostic accuracy using PCA3 during the initial prostate biopsy. In patients qualified for initial biopsy (PSA ≥ 4 and/or suspicious DRE), the Progensa test was not used, but the RC-PCR-based PCA3 test was used. The values obtained were sufficient to distinguish positive from negative prostate biopsy results but were not correlated with PCa aggressiveness.

In a study [25] (Table 2) involving Latino Americans, results were comparable to those obtained for other populations, indicating its potential use in Latino Americans with persistently elevated PSA and previous negative biopsies.

**Table 1:** Predictive capacity of prostate cancer after negative biopsy biomarkers.

Commercial Product	Biomarkers	Purpose	Indication	Cohort	Avoid Biopsies	Specimen	FDA Approved	Method	Predictive Capacity	Ref.
PCA3	lncRNA PCA3, PSA mRNA	Predicts the presence of malignancy. Supports initial biopsy decisions by enhancing diagnostic value. Determines whether repeat biopsy is needed after an initially negative biopsy.	Diagnosis: repeat biopsy Prognosis *	*n* = 233 [12] *n* = 859 [14] *n* = 1072 [19] *n* = 351 [26] *n* = 3073 [27]	For PCA3 score < 20 and PSA < 4 ng/mL 8% of men would have avoided biopsies, while 9% of cancer (non-HG) have been underdiagnosed. For only PCA3 score < 20 46% biopsies would have been avoided, while missing 12% of cancers (3% HG [14])	First catch (20–30 mL) post-DRE urine	Yes	Urine specimens were held at 2 °C to 8 °C and processed within 4 h by mixing with an equal volume of detergent-based stabilisation buffer (Gen-Probe® Hologic, San Diego, CA, USA) to lyse the prostate cells and stabilise the RNA. Samples were stored at −70 °C until testing and batch shipped on ice packs if needed. PCA3 and PSA mRNA were isolated from processed urine samples by capturing magnetic microparticles and amplified by transcription-assisted amplification. Products were detected with chemiluminescent DNA probes using a hybridisation protection assay [12]. Statistical analyses were performed by the data coordinating centre using SAS version 9.2 (SAS Institute, Cary, NC, USA) [14]. NCSS 2004 (NSCC Inc., Kaysville, UT, USA) was used for the analysis [19]. Data analysis was performed using Statistical Package for Social Sciences version 12.0.1 (SPSS, Chicago, IL, USA) [26].	AUC = 0.68 Se. = 58% Sp. = 72% [12] For detection of any cancer, PPV = 80% for initial prostate biopsy, and for repeat prostate biopsy NPV = 88% Se. = 76% Sp. = 52% [14] AUC = 0.693 Se. = 48.4% Sp. = 78.6% [19] AUC = 0.72 Se. = 61% Sp. = 74% [26] AUC = 0.697 for prediction PCa AUC = 0.682 for HG PCa NPV = 0.67 PPV = 0.62 Se. = 53% Sp. = 75% [27]	[12,14,19,26,27]
ConfirmMDx	Hypermethylation of GSTP1, APC and RASSF1 genes, PSA	Screening patients at risk of HG PCa after an initial negative biopsy. It is clinically validated for detection of PCa in tissue from PCa-negative biopsies. Helps to distinguish true negative biopsies from those with possible undetected cancer, and decide when to re-biopsy.	Diagnosis: repeat biopsy	*n* = 498 [28] *n* = 350 [29] *n* = 803 [30] *n* = 211 [31] **	30% of repeat biopsies can be safely avoided [30]	Tissue from prostate biopsy	No	All men underwent two consecutive biopsies: a negative index biopsy and then negative or positive rebiopsy. DNA was extracted and processed from fixed, paraffin-embedded blocks of prostate biopsy core tissue. In histologically negative prostate biopsy core tissues, epigenetic analyses were performed in a randomised, blinded fashion profiled for GSTP1, APC RASSF1 against the reference ACTB gene using methylation-specific PCR (MSP). In DOCUMENT (The Detection Of Cancer Using Methylated Events in Negative Tissue) [29] and studies [30,31] for direct comparison with the MATLOC (Methylation Analysis To Locate Occult Cancer) [28] cohort, the previously determined analytical gene cutoff values for determining methylation status were identical. All statistical analyses, including logistic regression and cross-validation, were performed in R software (R Foundation for Statistical Computing, Vienna, Austria).	NPV =90% Se. = 68% Sp. = 64% for any PCa [28] AUC = 0.628 NPV = 88% Se. = 62% Sp.= 64% [29] AUC = 0.742 NPV = 96% for HG NPV = 89.2% for all cancers PPV = 28.2% for any cancer [30] NPV = 78.8% PPV = 53.6% for detection of all PCa Se. = 74.1% Sp. = 60.0% [31] NPV = 94.2% PPV = 19.4% for detection of GS ≥ 7 PCa Se. = 77.8% Sp. = 52.7% [31]	[28,29,30,31]

*—Prognostic value of PCA3 is controversial. **—group of African Americans. Abbreviations: APC- adenomatous polyposis coli, ACTB-beta-actin (reference gene) AUC—area under the curve, GS—Gleason score, GSTP1- glutathione s-transferase pi 1, HG- high grade, lncRNA—long non-coding RNA, n—number of patients participating in study, mRNA—messenger RNA, NPV—negative predictive value, PCa—prostate cancer, PCA3—PCa antigen 3, PPV—positive predictive value, PSA—prostate-specific antigen, RASSF1—ras association domain family member 1, Ref—references, Se—sensitivity, Sp—specificity.

**Table 2:** Evaluation of biomarkers in multiethnic populations.

Commercial Product	African-Americans	Japanese Men	Chinese Men	Latino American
PCA3	In a study [21] on a racially diverse group of men, 60% of the participants were African-American. It demonstrated that the PCA3 test also in African-Americans improves the ability to predict the presence of any prostate cancer and high malignancy.	Study [22] examined the diagnostic utility of PCA 3 in Japanese men undergoing prostate biopsy. They achieved a similar diagnostic value to that obtained in men in Europe and the USA.	Studies [23,24] showed the utility of PCA3 in Chinese men.	In a study [25] involving Latino Americans, results were comparable to those obtained in other publications for other populations indicating its potential use in Latino Americans with persistently elevated PSA and previous negative biopsies.
ConfirmMDx	Study [31] showed no significant differences in sensitivity and specificity between this test and previously described validation studies involving predominantly Caucasian populations and indicates usefulness for African Americans in risk stratification after an initially negative biopsy.	No data about these ethnic groups were found.		
PHI	To assess the ability of PHI to detect Gleason grade 2-5 (GGG) PCa in African Americans, 158 patients with elevated PSA levels and 135 controls were recruited [32]. Results indicate that PHI ≥ 28.0 can be safely used to avoid unnecessary biopsies in African Americans.	In a study [33] involving a European (*n* = 503) and Asian (*n* = 1652) population, more biopsies were avoided in the Asian group (56% vs. 40%). This study also identified the need to establish differential cut-off points for diverse ethnic groups. The authors of the publication recommended cut-off points for csPCa: PHI > 40 for European men and PHI > 30 for men of Asian origin.		No data about these ethnic groups were found.
4Kscore	The study [34] included 366 men, 205 of whom were African American. The results of the study showed an AUC = 0.81 in predicting aggressive prostate cancer in this population, therefore the 4Kscore can be used to guide biopsy decisions also in this ethnic group.	A multiethnic group study [35] (African Americans, Japanese, Latinos, Native Hawaiians, and Whites) confirmed the 4Kscore′s accuracy to discriminate benign from malignant cases and indolent from aggressive tumors.		
Mi-Prostate Score	It is unknown what the cut-off values should be and what the diagnostic and prognostic accuracy. There is a lack of studies on African-American, Asian or Latino American populations.			
ExoDx Prostate IntelliScore	It is unknown what the cut-off values should be and what the diagnostic and prognostic accuracy. There is a lack of studies on African-American, Asian or Latino American populations.			
SelectMDx	It is unknown what the cut-off values should be and what the diagnostic and prognostic accuracy. There is a lack of studies on African-American, Asian or Latino American populations.			
Stockholm3 Model	This test was evaluated only on men from an ethnically homogeneous population (Stockholm County, Sweden).			

PCA3 shows more significant diagnostic and prognostic potential when combined with other biomarkers, such as TMPRSS2 fusion: ERG [36] hK2, PSA [37] and PSAD [38]. Currently, some researchers are making efforts to develop more precise detection methods for PCA3 [39,40].

### 3.2. Mi-Prostate Score (MiPS)

MyProstateScore (MPS, LynxDx, Ann Arbor, MI, USA (previously known as MiPS—Michigan Prostate Score)) is an algorithm that measures mRNA, PCA3 and TMPRSS-ERG (abnormal fusion of TMPRSS2 and ERG (T2-ERG)) expression in urine from the first collection after DRE and serum PSA.

More than 50% of patients with PCa have an ERG gene fusion with TMPRSS2 [41]. The presence of this translocation has been shown to be associated with poor patient prognosis—an increased risk of recurrence and mortality from PCa.

The test is indicated for men with suspicious PSA levels who are being considered for initial or repeat biopsy. The test result validates the need for biopsy and predicts the risk of high-grade prostate cancer (GS > 7) in a diagnostic needle biopsy [42,43,44]. Values range from 0 to 100, and the higher the score, the greater the risk of aggressive cancer.

In 2013, Salami et al. [44] showed that the MPS test was significantly more accurate than any single variable (TMPRSS2-ERG AUC = 0.77 compared with 0.65 for PCA3 and 0.72 for serum PSA alone), AUC was 0.88, with specificity and sensitivity of 90% and 80%, respectively (Table 3).

A pivotal study published by Tomlins et al. [43] in 2016 indicated the high diagnostic value of MPS, AUC = 0.751 for detecting PCa on biopsy and AUC = 0.772 for detecting clinically significant PCa (defined as Gleason ≥ 7), which was significantly better than for PSA alone (AUC = 0.651) (Table 3).

In a prospective study [45] involving 1077 men, MPS was shown to increase the detection of aggressive prostate cancers compared with PSA alone. When the cut-off point was set at 95% sensitivity, the specificity of detecting HG PCa increased from 18% (PSA alone) to 39%. The authors further demonstrated that if biopsies were performed in patients with positive urine PCA3 (score > 20) or T2-ERG (score > 8) or with serum PSA > 10 ng/mL, 42% of unnecessary biopsies could be avoided.

In a study including a validation cohort of 1525 men, the MPS test was confirmed to improve the detection of csPCa. The authors also intended to set a threshold to exclude GG cancer ≥ 2. An MPS threshold of ≤10 was recommended. At this value, sensitivity (96%) and NPV (97%) were obtained, avoiding 32% of unnecessary biopsies while missing 3.7% of GG cancer cases ≥ 2 (Table 3) [46].

### 3.3. ExoDx Prostate ^®^ (IntelliScore) (EPI)

The ExoDx Prostate (IntelliScore) (EPI, Exosome Diagnostics, Waltham, MA, USA) assesses the exosomal RNA expression of three genes (ERG, PCA3 and SPDEF) involved in the initiation and progression of PCa. ExoDx prostate is a test performed from a urine sample that does not require prior DRE testing. Exosomal RNA is derived from exosomes, which are small membrane vesicles secreted by several cell types, including immune and cancer cells [47]. The high potential of exosomes as biomarkers is due to their structure—a lipid bilayer protects the contents from degradation by proteases.

The test scores range from 1 to 100 and a cut-off point of 15.6 indicates men at increased risk of HG PCa (≥GG2) at subsequent biopsy, making the test helpful in validating the need for biopsy in men at risk. The test is recommended for men aged ≥50 years who are in the PSA “grey zone” (2–10 ng/mL) to distinguish a benign (HG1; when the test value < 15.6) from high-grade PCa (HG2 ≥ 15.6) [48].

**Table 3:** Predictive capacity of prostate cancer prebiopsy biomarkers.

Commercial Product	Biomarkers	Purpose	Indication	Cohort	Avoid Biopsies	Specimen	FDA Approved	Method	Predictive Capacity	Ref.
PHI	tPSA, fPSA, p2PSA	Estimates the probability of a diagnosis of all grades PCa and csPCa (GS ≥ 7). Indicates the need for a biopsy, reduces the number of unnecessary ones and continues to follow up. Reduces overdiagnosis and overtreatment.	Diagnosis: initial biopsy, repeat biopsy. Prognosis.	*n* = 893 [49]. Two independent cohorts *n* = 561 and *n* = 395 [50] *n* = 769 [51] *n* = 350 [52] *n* = 658 [53] *n* = 1652 Asian men and *n* = 503 European men [33] *n* = 531 [54] *n* = 16,762 [55].	A total of 26% of unnecessary biopsies [49]. In the primary cohort, avoided 41% of unnecessary biopsies. In the validation cohort, avoided 36% of unnecessary biopsies while missing only 2.5% of high-grade PCa [50] Among Asian men at 90% sensitivity for HG PCa and cut-off > 30, 56% of biopsies and 33% of GS 6 diagnoses could have been avoided [33]. Among European men at 90% sensitivity for HG PCa and cut-off > 40, 40% of biopsies and 31% of GS 6 diagnoses could have been avoided [33].	blood serum	Yes	Specimens were analysed at the EDRN Biomarker Reference Laboratory at Johns Hopkins University. Serum was stored at −80 °C before testing. Prebiopsy measurements of total PSA, fPSA and p2PSA were performed using an Access 2 automated immunoassay analyser (Beckman Coulter Inc, CA, US). Technologists performing the assays were blinded to prostate biopsy results. PHI was calculated using the equation (p2PSA/fPSA) × √(PSA) [50] Statistical analysis was conducted by using SAS, version 9.3 and R, version 3.1.0 (R Foundation for Statistical Computing, Vienna, Austria) [50].	AUC = 0.703 Se. = 95% Sp. = 16% [49] AUC = 0.815 for detecting aggressive PCa Se. = 95% Sp.= 36% [50] AUC = 0.73 [51] For prediction of GS ≥ 7 Se. = 90.8/66.3/44.8 Sp. = 34.8/66.3/89.9 for criterions ≥30.9/44.0/56.2, respectively [52] For prediction of GS 6–7 Se. = 89.9/60.0/37.4 Sp. = 26.0/61.6/90.4 for criterions ≥ 28.0/42.2/55.5, respectively [52] Se.= 80% for PCa/ csPCa and biopsy GS Sp. = 46.1/45.5/46.4, respectively [53] Se.= 95% of PHI for PCa/csPCa and biopsy GS Sp. = 14.1/16.3/27.4, respectively [53] AUC = 0.78 for PCa detection and for HG PCa (75% men were European) In Asian men group AUC = 0.76 for PCa detection and AUC = 0.77 for HG PCa Se. = 99–53% with corresponding Sp. = 10–72% in European group for cut-off = 25–55 [33] Se. = 96–27% with corresponding Sp. = 36–96% in Asian group for cut-off = 25–55 [33] AUC =0.704 for any cancer AUC = 0.711 for Gleason ≥ 7 [54] AUC = 0.76 for PCa detection Se. = 89% Sp. = 34% [55] AUC = 0.82 for HG PCa detection, Se. = 93% Sp. = 34% [55]	[33,49,50,51,52,53,54,55]
4Kscore	tPSA, fPSA, iPSA, hK2		Diagnosis: initial biopsy, repeat biopsy. Prognosis.	*n* = 531 [54] *n* = 16,762 [55] *n* = 1012 [56] *n* = 11,134 [57] *n* = 611 [58] *n* = 718 [59] *n* = 2872 [60]	Avoided 29% of biopsies, delayed diagnosis 10% of HG PCa [54] 43% avoided and delayed diagnosis of 2.4%. Gleason ≥ 7 for 9% 4Kscore cutoff [56]. 58% avoided and delayed diagnosis of 4.7% Gleason ≥ 7 for 15% 4Kscore cutoff [56]. reduction of 94.9%, 47.1% and 9.3% biopsies in men with low-risk, intermediate-risk and high-risk aggressive PCa, respectively [58] For different threshold 4%, 5%, 7.5%, 10% 58%, 66%, 75% and 80% of reduced biopsies while missed diagnose of HG PCa 1%, 2%, 2%, 2%, respectively [60]	Blood plasma	No	Blood was collected into tubes containing K2EDTA, inverted, centrifuged at 1600× *g* and frozen at 70 °C within 4 hours from collection. Frozen plasma was stored until shipped in dry ice to OPKO Labs (Nashville, TN, USA) for analysis. The analytical laboratory was blinded to all sample information and clinical data. Samples were thawed immediately before analysis. Then, tPSA, fPSA, iPSA and hK2 were measured. Statistical analysis was conducted by using R, version 3.1.1 (http://www.r-project.org/ accessed on 11 November 2016) [59].	AUC = 0.69 for any cancer AUC = 0.718 for Gleason ≥ 7 [54] AUC = 0.72 for PCa detection Se. = 74% Sp. = 60% [55] AUC = 0.81 for HG PCa detection Se. = 87% Sp. = 61% [55] AUC = 0.82 [56] AUC = 0.81 [57] AUC = 0.75 [59] AUC = 0.876 for 4Kscore AUC = 0.888 for 4Kscore with RPCRP Se. = 87% Sp. = 71% [60]	[54,55,56,57,58,59,60]
Mi-Prostate Score	PCA3 and T2-ERG mRNA, tPSA		Diagnosis: Initial Biopsy, repeat biopsy. Prognosis *	*n* = 497 [42] *n* = 1225 [43] *n* = 48 [44] *n* = 1077 [45] *n* = 1525 [46]	Total of 35% of biopsies and missing 13% of ≥GG2 PCa [42] avoided 35–47% of biopsies while delaying the diagnosis of 1.0–2.3% of ≥ GG2 [43]. Avoided 67% of biopsies at the risk of a false-negative rate of 20% [44]. Avoided 33% of unnecessary biopsies, missing 7% of HG PCa [45] for threshold ≤10; avoided 32% of unnecessary biopsies, missing 3.7% of GG ≥ 2 cancers [46]	Post-DRE first void urine	No	Urine samples were obtained immediately after DRE, refrigerated and processed within 4 h by mixing with an equal volume of urine transport medium and stored below −70 °C until analysis. The amount of T2: ERG and PSA mRNA was determined by TMA. Statistical analyses was performed using R version 2.10.1 (R Foundation for Statistical Computing, http://www.R-project.org accessed on 16 May 2015 [43]	AUC= 0.842 ** Se. = 88.1% Sp. = 49.6% [42] AUC = 0.772 [43] AUC= 0.88 for detection of PCa AUC= 0.772 for csPCa Se. = 80% Sp. = 90% [44] In the developmental cohort(*n* = 516) Se. = 95% Sp. = 39% In the validation cohort (*n* = 561) Se. = 93% Sp. = 33% [45] NPV = 97% Se.= 96% for threshold ≤10 [46]	[42,43,44,45,46]
ExoDx Prostate IntelliScore	Exosomal mRNA ERG, PCA3 and SPDEF		Diagnosis: initial biopsy, repeat biopsy. Prognosis *	Validation cohort *n* = 519 (training cohort *n* = 255) [48] *n* = 503 [61] *n* = 229 [62]	Total of 20% of all biopsies, 26% of unnecessary biopsies, and missing 7% of ≥GG2 PCa [61] 26% of all biopsies, 27% of unnecessary biopsies and 2.1% delayed detection of ≥ GG2 [62].	Urine	No ^1^	Urine samples were collected in a 15–20 mL container and stored at 4 °C. for up to 5 days before shipment to a central laboratory (Exosome Diagnostics, Inc., Waltham, MA, USA) for EPI assay analysis [61]. R software version 3.6.1 (R Core Team, 2019, Vienna, Austria) was used for reporting and data analysis. Two-tailed *p* values ≤ 0.05 were considered statistically significant. [63]	AUC = 0.73 (combined with SOC^2^) AUC = 0.71 NPV = 91% PPV = 36% Se. = 92% Sp. = 34% [48] AUC = 0.70 NPV = 89% Se. = 93% [61] AUC = 0.66 NPV = 92% Se. = 82% [62]	[48,61,62]
SelectMDx	HOXC6, KLK3, DLX1 mRNA and PSAd		Diagnosis: Initial Biopsy.	First cohort *n* = 519 Second *n* = 386 [64] *n* = 1955 [65] *n* = 172 [66]	Total of 42% of all, 53% of unnecessary biopsies [64].	Post-DRE first void urine	No	Approximately 30 mL of the first urine passed was collected into a collection cup after the DRE was performed. The urine was immediately transferred to a urine sample transport tube (Hologic San Diego, CA, USA) and samples were shipped at room temperature to a central laboratory and stored at −80 °C. Statistical analysis was performed using SPSS v.20.0 (IBM Corp., Armonk, NY, USA) and R v.3.2.1 (R Foundation for Statistical Computing, Vienna, Austria) [64].	AUC = 0.86 AUC= 0.90 (+ clinical parameters) NPV = 94% PPV= 27% Se. = 91% Sp. = 36% [64] AUC = 0.82–0.85 NPV = 95% Se. = 89–93% Sp. = 47–53% [65] AUC = 0.83 NPV = 98% Se. = 96% [66]	[64,65,66]
Stockholm3 Model ^3^	tPSA, fPSA, hK2, MIC1 and MSMB (with genetic markers based on (232–254 SNPs) ***		Diagnosis: Initial Biopsy.	Validation cohort = 111,819 (training cohort *n* = 32,453) [67] *n* = 59,159 [68] *n* = 533 [69] Two cohorts: *n* = 56,282 and *n* = 47,688 [70]	S3M could reduce the number of biopsies by 32% and could avoid 44% of benign biopsies [67], reduction in total biopsies 33–52% and avoid 42–62% of benign biopsies, while missing 10–20% GS ≥ 7 [68]. Total of 38% of all biopsy avoided, delaying diagnosis for 6% of men with GG ≥ 2 cancer [69] reduction in total biopsies 53% and avoided 76% of benign biopsies [70]	Blood plasma	No	Prior to prostate biopsy, sample blood was collected for testing. Biopsy results were used to validate the Stockholm3 test results.The program R version 3.4.2(R Foundation for Statistical Computing, http://www.R-project.org accessed on 31 August 2018) was used to perform the statistical analyses [69].	AUC = 0.69 for all prostate cancers AUC = 0.74 for Gleason ≥ 7 [67] AUC = 0.75 for Gleason ≥ 7 [68] AUC = 0.89 for GG ≥ 2 [69]	[67,68,69,70]

^1^—ExoDx received FDA Breakthrough Designation status in June 2019. Since October 2019, Medicare Administrative Contractor (MAC) National Government Services, Inc. has issued a final Local Coverage Decision (LCD) L37733, which covers the ExoDx Prostate test before an initial prostate biopsy. ^2^—SOC: prostate-specific antigen level, age, race, family history) ^3^—Predictors in S3m include prostate examination (DRE and prostate volume), clinical variables (first-degree family history of PCa, and a previous biopsy, age) *—Similar to PCA3, the utility for prognosis remains controversial. **—AUC for ERSPC risk calculator plus PCA3 plus TMPRSS2-ERG ***—The initial S3M version had also included intact PSA, but due to interference between kallikreins in the immunosorbent assay with the allergen chip, it was removed from the S3M. Recently, a novel biomarker, HOXB13 SNP, a rare germline mutation of the HOXB13 gene with a high impact on prostate cancer risk, was included. Abbreviations: AUC—area under the curve, csPCa—clinically significant prostate cancer, DLX1—distal-less homeobox 1, DRE—digital rectal exam, ERG—estrogen-regulated gene, fPSA—free PSA, GG—grade group, GS—Gleason score, HG—high grade, hK2—human kallikrein-related peptidase 2, HOXC6- homeobox C6, iPSA—intact PSA, KLK3—kallikrein-related peptidase 3, LG—low grade, *n*- number of patients participating in study, mRNA—messenger RNA, NPV—negative predictive value, PCa—prostate cancer, PHI—Prostate Health Index, PPV—positive predictive value, PSA—prostate-specific antigen, PSAd—PSA density, p2PSA—(−2) proPSA, Ref—references, SNPs—single-nucleotide polymorphisms, Se—sensitivity, Sp—specificity, STHLM3—Stockholm-3, SPDEF-SAM pointed domain-containing ETS transcription factor, T2-ERG—transmembrane protease serine 2-ERG, tPSA: total PSA, 4K—four-kallikrein panel.

A validation study [48] conducted in 2016 on a (training) cohort of 255 patients initially and a separate validation cohort of 519 patients tested the ability of the ExoDx test in combination with SOC (PSA level, age, race and family history) to identify PCa GS (Gleason score) ≥7 in men aged ≥50 awaiting their first biopsy (PSA 2–20 ng/mL and/or suspicious DRE). With a cut-off value of 15.6, ExoDx alone demonstrated a sensitivity of =91.89%, NPV = 91.3% and AUC of 0.71 for distinguishing GS ≥ 7from GS 6 and benign PCa. When the test was combined with SOC (AUC = 0.73), the ExoDx test outperformed SOC alone (AUC = 0.63) and the PCPTRC risk calculator (AUC = 0.62) in differentiating PCa GS ≥ 7od GS 6 and benign PCa.

In a study published 2 years later [61], McKiernan et al. evaluate the clinical utility of ExoDx in comparison with standard clinical parameters for distinguishing grade (GG) ≥ 2 PCa from GG1 PCa and benign disease (in men eligible for the first biopsy) and conducted a prospective study of 503 patients aged ≥50 years with PSA “grey zone” (2–10 ng/mL). The results obtained were similar to previous studies. The combined model of ExoDx and SOC achieved the highest value (AUC = 0.71), and ExoDx alone (AUC = 0.70) was better at predicting GG2 PCa at initial biopsy than SOC (AUC = 0.62). A value of 15.6 was confirmed as the recommended cut-off point to distinguish patients at high risk of GG2 PCa at their initial biopsy. At a cut-off point of 15.6, a high negative predictive value of NPV = 89% was achieved (Table 3), preventing 26% of unnecessary biopsies and 20% of all biopsies (with only 7% of ≥GS7 PCa missed).

These two prospective studies validating over 1000 patients [48,61] showed that ExoDx (AUC 0.71 and AUC 0.70, respectively) was better at predicting clinically significant PCa at first biopsy than existing risk calculators and PCPT-RC (AUC 0.63), ERSPC-RC (AUC 0.58) and PSA alone (AUC 0.58). Both studies show that this test is useful for risk stratification of ≥GG2 due to GG1 cancer and benign disease and improves identification of patients at higher risk of advanced prostate cancer and helps avoid unnecessary biopsies.

Other work [48,61] confirmed the utility of ExoDx for primary biopsy but lacked confirmation for use on repeat biopsy. McKiernan et al. conducted a study [62] in 229 patients qualified for repeat biopsy; an AUC of 0.66 and an NPV of 92% (irrespective of other clinical features) were achieved at a previously validated cut-off point of 15.6, which would avoid 26% of unnecessary biopsies, omitting only 2.1% of patients with HG PCa (Table 3). Furthermore, in this study, AUC curves and net health benefits analyses showed better performance of ExoDx than the ERSPC and PSA risk calculator in predicting HG-PCa in men with a prior negative prostate biopsy. A total of 71.6% of patients were Caucasian, 14.4% African American and the study was completed on the most ethnically diverse group. The vast majority of publications are from the USA, and no studies have been completed on Asian or African populations or, more widely, on African Americans. It is not known what the cut-off values should be and what the diagnostic and prognostic accuracy is for a multiethnic population.

The clinical utility of ExoDx Prostate was recently evaluated in 1094 patients scheduled for their first biopsy (with PSA 2–10 ng/mL). This first study [63] of PCa biomarkers with a blinded control arm showed that ExoDx helped avoid unnecessary biopsies when the test was negative and increased the detection of HG PCa by 30% compared with a control arm without ExoDx (SOC alone). Compared to SOC, the test missed 49% fewer HG PCa. The study showed that ExoDx improved patient stratification and influenced the decisions made by (68%) urologists about biopsy (with rising PSA being the main reason for not following ExoDx results).

### 3.4. SelectMDx

SelectMDx (MDxHealth, Inc., Irvine, CA, USA) is a urine-based test after DRE that measures three biomarkers: DLX1 (progression gene), HOXC6 (cell proliferation gene), KLK3 (reference gene) and clinical risk factors (age, DRE, PSA and prostate volume, which can be calculated from the TRUS measurements substituted into the formula: height × width × length × 0.523). HOXC6 and DLX1 mRNA levels are assessed to estimate the risk of PCa on biopsy and the presence of high-risk cancer.

Men with elevated PSA levels in the “grey zone” (4–10 ng/mL) and/or an abnormal DRE result are subjects for whom an initial biopsy is considered. The result determines whether the patient is at high or low risk of PCa. It supports clinical decision-making and stratifies patients into those who may benefit from biopsy and early cancer detection and others for whom it is better to avoid this invasive procedure and continue with routine screening or active surveillance.

In a study [64], 386 men with an elevated PSA (≥3 ng/mL), abnormal DRE or family history of PCa, awaiting initial or repeat biopsy were studied. The predictive model (which included DRE as an additional risk factor) achieved an AUC = 0.86 in predicting high-grade cancer (after biopsy). Moreover, it was shown that with a cut-off point of −2.8, a 98% NPV with a sensitivity of 96%, the risk of GS ≥ 7 PCa was very low. For GS = 7 PCa, a 53% reduction in unnecessary biopsies was achieved while missing only 2% of cases with csPCa.

A study [65] was performed on a multinational (Netherlands, France, Germany) group of 715 patients with PSA < 10 ng/mL, before the initial prostate biopsy. SelectMDx achieved very high predictive values (AUC = 0.82 with 89% sensitivity, 53% specificity and NPV = 95% (Table 3), outperforming the PCPTRC 2.0. risk calculator (AUC = 0.70). This supports the use of the SelectMDx (MDxHealth, Inc., Irvine, CA, USA) test for the detection of HG PCa prior to the initial prostate biopsy.

To evaluate the clinical utility of SelectMDx, 418 patients who had an initial biopsy were studied. A total of 165 of them were positive. The number of biopsies performed within 3 months of the test was reviewed. For patients with a positive result, 71 patients (43%) were biopsied—27 of these patients were identified as having cancer, including 10 with a grade > 2. During this time, 9 patients with negative SelectMDx test results (3.6%) were biopsied—4 were identified as having cancer—all with a grade ≤ 2. SelectMDx has been shown to have a significant impact on decisions about the frequency and timing of biopsies. When the test was positive, the time period was shorter (median: 2 months) and the number of biopsies was five times higher than when SelectMDx was negative (median: 5 months). The test assisted urologists in their decision-making and is, therefore, a useful tool in daily urological practice [71].

A typical dilemma for the urologist deciding on a repeat prostate biopsy was presented in a case report of two men [72]. Both patients had already had their first negative biopsy, with normal DRE results, serum PSA levels of 3–10 ng/mL, no family history of PCa (and a negative ERSPC RC4 risk score). In these considerations, the European Association of Urology (EAU) recommendations [4] suggest the inclusion of mpMRI, RC and/or liquid biopsy tests. The mpMRI is the most accurate tool for localisation of PCa, but this imaging modality performed in the second patient did not show the presence of a tumour. The SelectMDx test showed the presence of PCa and therefore played a key role in individualising the need for repeat biopsy. In the mentioned report, NPV = 98%, and the risk score correlates with the mpMRI results, but it describes only two cases; therefore it suggests and indicates the need for further studies in risk stratification for repeat biopsy using the SelectMDx test.

It is unknown what the cut-off values should be and what the diagnostic and prognostic accuracy is for a multi-ethnic population. There is a lack of studies on Asian or African American populations.

## 4. Serum Biomarkers

Serum biomarkers, determined from blood samples, are produced by healthy and abnormal cells. PSA is undoubtedly the most widely studied cancer biomarker, but its clinical utility due to low specificity and specificity raises the need to find a test with better diagnostic values. Three tests that may have a positive impact on clinical practice are described below.

### 4.1. Prostate Health Index (PHI)

The Prostate Health Index (PHI; Beckman Coulter Inc., Brea, CA, USA), determined from a serum test, includes total PSA (tPSA), free, non-protein-bound PSA (fPSA) and (–2)proPSA (the fPSA isoform resulting from incomplete processing of the PSA precursor).

Determination of PHI values is indicated in men with PSA levels in the “grey zone” (4–10 ng /mL) and an unsuspected digital rectal examination (DRE) result [53], at age ≥50 years. The PHI score is calculated from the formula: ((–2)proPSA/fPSA) × √PSA.

The above formula indicates that men with lower fPSA, higher total PSA and (–2)proPSA are at an increased risk of development of clinically significant PCa [73].

This was confirmed by a study [74] in which the authors demonstrated that a low fPSA with a high total PSA indicates a risk of more aggressive PCa.

High PHI values indicate an increased likelihood of detecting prostate cancer, so when a biopsy (initial or repeat) is recommended, consideration should be given to using this less invasive method.

The PHI score has a greater diagnostic value than considering each of the indices (tPSA, fPSA) separately [49,51], improves the detection of PCa [75], improves clinical decision-making and predicts PCa aggressiveness [49,76,77]. Although the PHI score mainly provides information on overall PCa risk, studies [49,53] show an association between PHI value and prediction of PCa GS ≥ 7. A study [49] reported AUC = 0.72 to distinguish PCa GS ≥ 4 + 3 from GS ≤ 3 + 4 or no PCa.

Teams of researchers Lepor et al. [78] and Loeb et al. [53] showed that PHI is more specific in detecting csPCa than tPSA and/or fPSA. Furthermore, they concluded that this test might be useful in active surveillance and prediction of adverse outcomes after prostatectomy. Guazzoni et al. [52] showed that this was due to (–2)proPSA, as at GS ≥7, both PHI and (–2)proPSA were significantly elevated.

De la Calle et al., based on a multicentre study [50], showed that PHI is a predictor of PCa GS ≥ 7 (AUC = 0.78–0.82). When the PHI cut-off value of 24 is taken, 36% of unnecessary biopsies are avoided, while only 2.5% of high-grade cancers are missed. With a PHI cut-off point of 25, 40% of biopsies would be missed, and detection of lower grade PCa cases (GS = 6) would be reduced by 25%. However, this is associated with an underdetection of approximately 5% of clinically significant cancer cases.

A study [79] also found a significant effect of PHI on biopsy decisions. The study included 506 men diagnosed using PHI score and 683 without PHI determination, who were the control group. In both groups, men had PSA in the range of 4–10 ng/mL and unsuspecting DRE results. PHI score influenced medical management in 73% of patients; when the score was low, biopsy was postponed, and when it was high or moderate (PHI ≥ 36), biopsy was performed. Men who had a PHI test had fewer biopsies than the control group: 36.4% vs. 60.3%, respectively.

In response to these publications, Ehdaie and Carlsson [80] expressed concern about excluding men from a biopsy on the basis of PHI values and the risk of overlooking aggressive cancer, pointing out that the rate of an omitted PCa was 30%.

The authors of the paper [79], in response [81] to [80], maintain that the biopsy was safely postponed. They cite NCCN and AUA recommendations that men without biopsy who are in the diagnostic grey area will be monitored more closely or with additional methods. In a second response [82], it was shown that due to the small number of high-grade cancers, the study would not allow drawing firm conclusions.

In a study [33] (Table 2) involving a European (*n* = 503) and an Asian (*n* = 1652) population, the use of PHI established the recommended cut-off points for the above ethnic groups. More biopsies were avoided in the Asian group (56% vs. 40%). This study also identified the need to establish differential cut-off points for different ethnic groups. The authors of the publication recommended cut-off points for csPCa: PHI > 40 for European men and PHI > 30 for men of Asian origin. This result is not surprising, as Asians have a four times lower risk of prostate cancer than Europeans.

To assess the ability of PHI to detect Gleason grade 2–5 (GGG) PCa in Black men, 158 patients with elevated PSA levels and 135 controls were recruited [32] (Table 2). With PSA ≥ 4.0 and PHI ≥ 35.0, 33.0% of unnecessary biopsies were avoided, but 17.3% of GGG 2–5 PCa were missed. With PSA ≥ 4.0 and PHI ≥ 28.0, 17.9% were avoided, and the sensitivity of detecting GGG 2–5 PCa was 90.4%. These results indicate that PHI ≥ 28.0 can be safely used to avoid unnecessary biopsies in Black men, although it is associated with a risk of missed detection of GGG 2–5 PCa.

Currently, some researchers are considering the use of the PHI density index (PHID—calculated as PHI divided by prostate volume) in diagnosis to identify csPCa. Tosoian et al. [83] showed that the prevalence of csPCa is associated with higher PHID and has a higher diagnostic value compared to PHI (AUC= 0.84 vs. 0.76). Their study indicates that PHID can prevent 38% of unnecessary biopsies while failing to detect only 2% of csPCa.

Another article [84] examined the diagnostic efficacy of PHI and PHID in terms of avoiding unnecessary biopsies. The results indicate that PHI (AUC = 0.722) and PHID (AUC = 0.739) have a higher diagnostic value than PSA, f-PSA% and PSAD (AUC = 0.595, 0.612 and 0.698, respectively). The combined sensitivity of PHI and PHID was 98%, avoiding 20% of biopsies in the non-diagnosis of only one patient with csPCa. Therefore, the use of the PHID density index may be a promising tool in the evaluation of PCa.

### 4.2. 4Kscore

The 4Kscore (Opko Health Inc., Miami, FL, USA) is a test developed to identify HG-PCa in patients with a suspicious DRE or elevated serum PSA. The test measures the levels of four kallikreins (4K): total PSA (tPSA), free PSA (fPSA), intact PSA (iPSA) and serum levels of human kallikrein 2 (hK2). It then compares the values obtained in the algorithm with the patient’s age, DRE and results of previous prostate biopsies. Based on this information, the algorithm generates a percentage probability score to predict HG PCa even years in advance. This assessment allows further management to be determined depending on the outcome of the test and a decision to perform an initial or subsequent biopsy. This test is recommended primarily for men with a genetic family history. However, it can be performed by any man over 35 years of age who wants to assess his personalised risk of disease in the future.

The 4K test, although not designed to assess the predicted course of already diagnosed prostate cancer, has also been used in patients with csPCa to identify candidates for more intensive therapy. It has also been used to improve treatment selection and thus increase the chance of cure in patients suspected of having an underestimated malignancy. The 4Kscore provides an estimate of a patient’s risk of developing distant metastases within 10 years.

Parekh and colleagues [56] on a validation cohort of 1012 indicated that the 4Kscore was better at predicting clinically significant PCa than the Prostate Cancer Prevention Trial Risk Calculator 2.0 (PCPT RC) (AUC 0.82 vs. 0.74) (Table 3). This study also indicates that, depending on the cut-off point, 30–58% of biopsies were reducible, while missing only 1.3–4.7% of HG PCa. A threshold of 1–7.5% is considered low risk, allowing safe delay of biopsy and continued follow-up with PSA. A cut-off of 9% reduces the number of biopsies to 43%, with 2.4% of csPCa cases missed [56,58]. At a cut-off of 15%, this test avoids prostate biopsies performed for indolent cancer by up to 58% and misses 4.7% [56]. A cut-off score of ≥20% indicates the need for biopsy due to the high risk of csPCa.

A comprehensive systematic review [57] including 12 studies (11,134 patients) showed, almost identically to the above study, an AUC = 0.81 for the 4Kscore in detecting csPCa (Table 3).

In a study [85], 43,692 asymptomatic men (unscreened, PCa-free, with low PSA values) were followed for 20 years, and the 4Kscore was evaluated for early detection of malignant prostate cancer. This work aimed to estimate the risk of prostate cancer metastasis or death by analysing the 4Kscore and PSA. It turned out that already at the time of blood collection, the 4Kscore indicated in whom an aggressive form of prostate cancer would appear. The 4Kscore significantly improved the detection of HG PCa in men with moderately elevated PSA. The authors concluded that men with an elevated PSA but a low 4Kscore could be safely observed by performing blood marker tests instead of direct biopsy. They indicated that men with a low 4Kscore have a very low long-term risk of death from prostate cancer or metastasis.

In a study [58] involving 611 patients, the 4Kscore test was ordered to assess the risk of aggressive prostate cancer in men with abnormal PSA and/or DRE results. Patients were divided into three risk groups: low, medium and high. The test results influenced biopsy decisions in 88.7% of men, where the biopsy avoidance rates were: 94.0%, 52.9% and 19.0% for the low, intermediate and high-risk groups, respectively. The risk category assessed by the 4Kscore was closely related to biopsy outcome, confirming the usefulness of the test in clinical practice.

A case–control study [35] (Table 2) evaluated the 4Kscore in 1667 prostate cancer cases and 691 control men with PSA ≥2 ng/mL. The men were from a variety of ethnic groups, including African American, Hispanic, Japanese, Native Hawaiian and Caucasian men. Results showed that across all ethnic groups, the 4Kscore was better at detecting both general and aggressive prostate cancer than tPSA or tPSA + fPSA. Therefore, the 4Kscore has broad clinical applicability and can be used for prostate cancer screening in a multiethnic population.

A study [56] conducted in the USA, evaluating the efficacy of the 4Kscore, examined 1012 men scheduled for prostate biopsy. The diagnostic performance in detecting HG-PCa was evaluated, showing an AUC of 0.82. African American (AA) patients comprised only 8.1% of the study group, which meant that the results were not representative of AA. For this reason, a validation study [34] (Table 2) was conducted on a population with a higher proportion of AA patients. The study included 366 men, 205 of whom were African American. The results of the study showed no significant difference in predicting tumour aggressiveness in this population, showing AUC = 0.81; therefore, the 4Kscore can be used to make biopsy decisions in both African Americans and non-African Americans.

A study [60] aimed at reducing unnecessary biopsies and overdiagnosis of benign PCa used the 4Kscore and the RPCRP risk calculator to predict csPCa at biopsy. A study of 2873 men showed that RPCRP and 4Kscore had very similar performance (AUC = 0.868 vs. AUC = 0.876), and their combination gave even better results (AUC = 0.888). This indicates that adding further predictors is a compromise between clinical utility, cost and patient burden.

### 4.3. Stockholm3 Model

Stockholm 3 Model (S3M) combines serum biomarkers (total PSA, free PSA, free/total PSA ratio, hK2, MIC1 and MSMB with genetic markers (254 single nucleotide polymorphisms [SNPs] and an unclassified variable for SNP HOXB13). The test also takes into account clinical data (age, previous prostate biopsy—family history, use of 5-alpha reductase inhibitors) and prostate examination (DRE, prostate volume). The S3M available in Sweden, Norway, Denmark and Finland is in clinical use for predicting the risk of aggressive prostate cancer and assessing the need for biopsy. The S3M research team at Karolinska Institute is currently working with two major laboratories in Europe, as well as laboratories in the US and Canada, to introduce the test in additional countries around the world. Additional validation studies have been conducted in Germany, the Netherlands and the UK. Studies on non-Caucasian populations (e.g., Hispanics, African Americans, Asians) are also planned. If the S3M is negative, the man has a low or normal risk of prostate cancer and is recommended to be followed up in 6 years. If the test is positive, it is recommended that the man is referred to a urologist. The urologist measures the volume of the prostate gland and carries out a DRE. If the prostate volume and/or the DRE test is abnormal, a biopsy is recommended. Otherwise, a Stockholm3 test in 2 years is recommended.

A study involving 59,159 men [67] compared S3M with PSA ≥ 3 ng/mL, a screening test for prostate cancer. The study was designed so that both tests detected the same number of Gleason score (GS) ≥ 7 tumours, and the tests were graded on the number of biopsies needed to achieve this. The results showed that S3M, in detecting tumours with a Gleason score of at least 7, has significantly higher specificity, sensitivity and AUC (0.74 versus 0.56 for PSA) for csPCa. Patients with a S3M score ≥ 11% were recommended to be referred to a urologist for further diagnosis. With a retained GS sensitivity ≥7, S3M avoided 32% of prostate biopsies (Table 3). In benign tumours, the level of biopsies avoided was 44%. In addition, the authors indicated that S3M could detect aggressive cancer even in men with PSA levels of 1.5–3 ng/mL, and the number of tumours with a Gleason score ≤6 was reduced by 17%, reducing overdiagnosis.

A study [68] described how, after fitting S3M to more data, the updated S3M slightly improved the AUC in predicting prostate cancer GS ≥ 7 compared with previously published results [67] (0.75 vs. 0.74). Each additional predictor (including DRE, previous biopsies and prostate volume) increased the AUC by up to one unit. The combination of predictors helps to increase the accuracy of diagnosis while reducing the number of unnecessary biopsies.

Studies [67,68,69,70,86] prove that S3M reduces overdiagnosis and the number of prostate biopsies while maintaining sensitivity for clinically significant prostate cancer.

In a short report [86], the authors evaluated how the S3M threshold affects the number of cancers detected and the number of biopsies performed. They collected data from a validation cohort of 47,688 men (with PSA ≥ 1 ng/mL) and then calculated the percentage of biopsies avoided and the percentage of cancer detections for different cut-off points of the S3M test. They noted that as the cut-off point increased, the number of cancers detected and biopsies performed decreased. They considered it reasonable to use S3M test values between 7% and 14% for the cut-off point for biopsy decisions, where cut-off values below 10% would increase sensitivity for Gleason score tumours ≥ 7 compared with PSA ≥ 3 ng/mL. They noted that the threshold could be selected to fit different health systems and even individual men.

Long-term follow-up of the replacement of PSA (as part of the standard prostate cancer diagnostic procedure) with Stockholm3 in prostate cancer detection in primary care in the Stavanger region of Norway showed that the implementation was beneficial. Compared with PSA, S3M reduced the proportion of clinically insignificant PCa (from 58% to 35%) and the number of biopsies performed (from 29.0% to 20.8%). In addition, it increased the proportion of biopsies positive for csPCa from 42% to 65%. This management may also lead to a reduction in healthcare costs. It has been estimated that direct healthcare costs decreased by 23–28% per male studied [87].

S3M is not suitable for men who have previously been diagnosed with or treated for prostate cancer or who are under follow-up after prostate cancer. It has no proven value for men diagnosed with prostate cancer or who have undergone a biopsy or other examination by a urologist within the last 6 months. It does not replace biopsy in men under active monitoring. This test was not evaluated on men younger than 50 years or older than 70 years and was restricted to an ethnically homogeneous population (Stockholm County, Sweden). The S3M was shown to be superior to prostate-specific antigen (PSA) as a screening tool for prostate cancer in all men aged 50–70 years. Furthermore, the S3M test can be performed in cases where the PSA value is > 1.5. The S3M has been shown to be superior in detecting, now often overlooked, aggressive cancer in men with PSA levels of 1.5–3 ng/mL. The S3M may reduce unnecessary biopsies without compromising the ability to diagnose prostate cancer with a Gleason score of at least 7.

## 5. Tissue Biomarkers

Tissue biomarkers analyse changes in nucleic acid expression and composition of tissue collected during needle core biopsy of the prostate. The main concept is to detect changes in the histologically normal field neighbouring prostate cancer. This helps to verify whether the patient requires an additional biopsy.

### ConfirmMDx (MDxHealth)

ConfirmMDx (MDxHealth, Inc., Irvine, CA, USA) is a tissue-based epigenetic assay that uses methylation-specific PCR (MSP) to analyse three prostate-cancer-related changes in DNA methylation patterns of suppressor genes (GSTP1, APC and RASSF1) in biopsy tissue (formalin-fixed and paraffin-embedded). All these biomarkers were isolated from biopsy-positive tissue.

ConfirmMDx is a molecular test clinically validated for predicting prostate cancer risk in men who have had a traditional thick-needle prostate biopsy that did not reveal the presence of cancer cells in collected histopathological material. In many cases, prostate biopsy results are falsely negative. The biopsy specimen may not be cancerous, and the histopathological result will not reveal the presence of cancer. However, due to the “halo effect”, tissue with a normal morphological appearance will show epigenetic changes, indicating the presence of cancer. Using the test, histopathological material that has already been taken—during a prostate biopsy—can be re-examined in a detailed epigenetic analysis quantifying the level of methylation of promoter regions of three genes in benign prostate tissue, assessing with high accuracy the presence of cancer cells in neighbouring areas.

ConfirmMDx offers the opportunity to avoid unnecessary repeat biopsies. It allows a decision to be made on whether to include (rule-in) or exclude (rule-out) therapy. High-risk men with a previously negative biopsy may have undetected cancer after the test. Such patients with a previous “false negative” biopsy result should be included for repeat biopsy and appropriate treatment.

It also allows low-risk men to be excluded from repeat biopsies, which protects the patient from unnecessary stress and possible complications and reduces healthcare costs. This test increases the negative predictive value.

In the MATLOC study [28] involving 498 men with histopathologically negative prostate biopsies who had repeat biopsies within 30 months, positive results (cases) and negative results (controls) were reported. The clinical impact of a panel of epigenetic markers was assessed, showing for all cancers: NPV, sensitivity and specificity of 90%, 68% and 64%, respectively (Table 1). The results showed that in a multivariate model including patient age, PSA, DRE and histopathological features of the first biopsy, the epigenetic test was a significant independent predictor. At the same time, it was shown that the addition of this test could improve the diagnostic process for prostate cancer and reduce the number of unnecessary biopsies.

This was confirmed in the multicentre DOCUMENT study [29], which validated the clinical ability of ConfirmMDx to predict negative histopathological results in repeat prostate biopsies. For this purpose, archived core tissue samples from prostate biopsies with negative prostate cancer from 350 patients were evaluated. All patients had repeat biopsies after 24 months with negative (control) or positive (cases) histopathological results. The epigenetic test was shown to be a significant independent predictor of PCa detection after repeat biopsy and showed an NPV of 88%, with a sensitivity = 62% and specificity= 64% (Table 1).

Van Neste et al. [30] conducted a study on a cohort of 803 men, stratified according to their general methylation status (positive or negative) as defined in MATLOC [28] and DOCUMENT [29]. This study demonstrated an NPV of 96% for csPCa, and that methylation intensity was strongly correlated with the cancer stage. In assessing the prediction of GS ≥ 7 PCa after repeat biopsy, ConfirmMDx reached an AUC = 0.76 (Table 1). The decision curve analysis indicated the high clinical utility of the risk score as a decision tool in repeat biopsy. This indicates that ConfirmMDx is a much better predictor compared to currently used indicators such as PSA and risk calculator (PCPT-RC).

A population of 211 African American men undergoing repeat biopsy was studied to compare the accuracy of predicting repeat biopsy outcomes with previous studies conducted in predominantly Caucasian populations [31] (Table 2). The specificity of this epigenetic assay was 60.0% and the sensitivity was 74.1% for detecting PCa at repeat biopsy. For detection of all PCa and GS ≥ 7 PCa, the NPV was 78.8% and 94.2%, respectively (Table 1). This study showed no significant differences in sensitivity and specificity between this test and previously described validation studies involving predominantly Caucasian populations and indicates usefulness for African Americans in risk stratification after an initially negative biopsy.

Wojno et al. [88] in 2014 already noted a reduced number of biopsies in clinical practice in centres using ConfirmMDx. They studied 138 patients with a median PSA level of 4.7 ng/mL and previous negative biopsies. They indicated a 4.4% repeat biopsy rate in ConfirmMDx-negative men, compared with a 43% prior repeat biopsy rate, indicating a potential 10-fold reduction.

A later study [89] in 2019 confirmed the impact of ConfirmMDx on biopsy decision-making. A total of 605 men with a median PSA level of 6.8 ng/mL and previous negative biopsies were studied. There was a six times higher repeat biopsy rate in ConfirmMDx positive men than in men with a negative test result. 

ConfirmMDx enables a higher degree of accuracy (previously unattainable by prostate biopsy procedures alone) and has clinical, financial [90] and health benefits by reducing the number of medically unnecessary and expensive repeat biopsies that are part of the current standard of care.

## 6. The Financial Aspect

A prostate MRI costs between 500 and 2500 USD in the United States, depending on whether the patient is insured. Approximately 1 million American men are currently referred for prostate biopsy each year. If all of these men underwent an MRI instead, costs could reach 3 billion USD per year.

In a paper [91] addressing the costs associated with prostate biopsy and its potential complications, the authors analysed charges for the procedure and related claims for all Medicare Fee-for-Service patients over a 2-year period (January 2014–December 2015). The study included 234,819 prostate biopsy cases and associated costs.

Uncomplicated biopsies cost about 1750 USD, those with one complication were already more expensive at 4060 USD, and for patients requiring hospital admission, the cost was as high as 13,840 USD (average cost was 2020 USD). The most common complication of biopsy is bleeding and infection, which can be prevented using biomarker tests from urine or blood. The cost of tests based on these is higher than the commonly used PSA but lower than biopsy, which makes it a cost-effective option.

In a paper [92], Santhianathen et al. conducted a cost-effectiveness analysis of biomarkers for 2018. Costs were obtained directly from pharmaceutical companies (these were as reported by Prostate Cancer Markers): PHI 499 USD, 4Kscore 1185 USD, SelectMDx 500 USD and ExoDx 760 USD the cost of ExoDx was estimated using data from the CMS (Centers for Medicare and Medicaid Services) Clinical Laboratory Fee Schedule). Discounted QALYs and costs were estimated; for example, a 50-year-old male with an elevated PSA level (3 ng/mL or greater). The cost of the current SOC strategy of ultrasound-guided transrectal biopsy was 3863 USD and the discounted QALY (an indicator of an individual’s or group’s health status expressing quality-adjusted life expectancy) was 18.0853. Each of the biomarkers tested improved the QALY compared with SOC. The ExoDx index provided the highest QALY with an incremental cost-effectiveness ratio of 58,404 USD per QALY. The study showed that before biopsy in men with elevated PSA levels, the use of SelectMDx (MDxHealth, Inc., Irvine, CA, USA) or EPI (Exosome Diagnostics, Waltham, MA, USA) assesses were cost-effective, PHI (Beckman Coulter Inc., Brea, CA, USA), was found to be more expensive and less efficient.

In an economic evaluation, Nicholson et al. [93], comparing diagnostic value for money, found that the PHI test and PCA3 were no more cost-effective than clinical evaluation, which also generates more QALYs.

Reports [94] on SelectMDx support data from four European countries [95], which showed that SelectMDx in the initial diagnosis of prostate cancer saves healthcare costs and increases QALYs compared with the current standard of care based on prostate biopsy for elevated prostate-specific antigen [95].

This was confirmed in a study by Govers et al. [96] on a population of US men with elevated PSA. Results were related to QALYs and cost of care from a payer (Medicare) perspective. Routine use of SelectMDx to guide biopsy decisions was shown to be beneficial—gaining an average of 0.045 QALYs and saving 1694 USD per patient.

Based on studies [92,95,96], it can be concluded that a SelectMDx-based strategy improves health outcomes and reduces costs.

The publication [90] focused on determining the impact of ConfirmMDx on the healthcare budget. It examined whether costs are recovered by avoiding unnecessary biopsies.

The implementation of ConfirmMDx created a hypothetical commercial health plan in which direct costs were calculated over a 1-year horizon using 2013 Medicare fee-for-service rates. The study concluded that the net cost of a commercial health plan with 1 million members would be reduced by approximately 500,000 USD if patients with histopathologically negative biopsies were screened using an epigenetic test to distinguish between patients who should undergo repeat biopsies and those who should not. The use of this genetic test may reduce healthcare costs and improve clinical management.

STHLM3 is not a commercially available test, for which reason its price is unknown. However, it is expected to be similar to other biomarkers currently available (>224 USD). These tests are more expensive than the common PSA, but are more reliable and can be performed less frequently due to their better diagnostic value. It also avoids biopsies, reduces overdiagnosis and allows a treatment plan to be customised to the patient and thus also reduces costs.

## 7. Guidelines

The National Comprehensive Cancer Network (NCCN) guidelines—version 2.2020—recommend considering the use of biomarkers for the early detection of prostate cancer, indicating that the specificity of screening can be improved in assessing the indication for biopsy (Grade C recommendation). They indicate the possibility of using the Prostate Health Index (PHI), SelectMDx, 4Kscore and ExoDx to assess the likelihood of high-grade cancer (Gleason score ≥ 3 + 4, GG ≥ 2).

The NCCN guidelines also address post-biopsy management. They indicate the possibility of using tests to improve specificity in high-risk patients despite a negative prostate biopsy result: 4Kscore, PHI, percentage free PSA, PCA3 and ConfirmMDx (included/added from 2020). The recommendations for the management of benign biopsy results themselves have changed “PSA and DRE 6–24 months apart and consideration of per cent free PSA, 4Kscore, PHI, PCA3 or ConfirmMDx and/or mpMRI and/or improved prostate biopsy techniques. Repeat prostate biopsy, depending on risk”. However, the guidelines note that the extent to which tests are validated in different populations varies and that it is unclear what the optimal combination of tests with MRI would be. In the current NCCN guidelines, MPS is listed as a biomarker requiring additional testing.

The EAU gives a strong recommendation for the use of risk-calculators and imaging in asymptomatic men with PSA levels of 2–10 ng/mL, while giving a weak recommendation (strength rating—weak) for urine and blood biomarkers to avoid biopsy [4].

The FDA has approved PCA3 and PHI. ExoDx received FDA Breakthrough Design recognition in June 2019. SelectMDx has not been reviewed by the FDA due to the agency determining that such approval is not necessary but includes CAP (College of American Pathologists) and CLIA (Clinical Laboratory Improvement Amendments) accreditations.

ConfirmMDx and 4Kscore do not have FDA recommendations but are accredited by CAP and CLIA.

## 8. Biomarkers and mpMRI

Multiparametric magnetic resonance imaging (mpMRI) is a promising new tool for the diagnosis, prognosis and monitoring of PCa.

The European Society of Urology guidelines on PCa recommends the use of mpMRI before prostate biopsy in previously untreated patients with suspected PCa [4].

In addition to the high sensitivity of mpMRI in detecting hg-PCa, mpMRI also has disadvantages, i.e., low specificity, high cost, the need for expensive, specialised equipment, low sensitivity in predicting the presence of extra-urethral expansion and the requirement for an expert review. Current research focuses on comparing biomarker tests with mpMRI and also on the extent to which they can complement each other.

A study [66] on 172 men showed promising correlation results between SelectMDx and mpMRI. There was a statistically significant difference in the SelectMDx score between PI-RADS 3 and 4 (*p* < 0.01) and between PI-RADS 4 and 5. The SelectMDx score was better than the PCA3 score in predicting outcome for suspected PCa on mpMRI (AUC = 0.83 for SelectMDx versus 0.65 for PCA3), suggesting the possibility of using the SelectMDx test to stratify patients for mpMRI.

The combination of 4KScore (AUC = 0.70) with mpMRI (AUC = 0.74) resulted in a prognostic improvement (AUC = 0.82 for 4KScore and mpMRI combined) in detecting aggressive PCa [97,98].

In a recent article [94], the authors demonstrated that the 4KScore, used in addition to mpMRI, can reduce unnecessary SBx (without worsening the diagnosis of csPCa) and identify patients who would benefit from undergoing TBx alone. An evaluation of 408 men showed a reduction (39.5%) in unnecessary biopsies and a reduction in detection (33.9%) of GG1 disease, with 5.2% (diagnosed with SBx) and 1.1% (diagnosed with SBx combined with TBx) missing.

In another study [99], 266 men who were not biopsied underwent three strategies using 4Kscore, mpMRI and combination PSA density (PSAD) to determine the safest method to skip biopsy. The first strategy starts by assessing the 4Kscore value. If it was >7.5, indicating an intermediate or high risk of csPCa, mpMRI was performed. If it was negative and the 4Kscore value was above 7.5 but below 18 (intermediate risk), the patient remained under clinical observation, but in case of a positive mpMRI result, a biopsy was performed. The second strategy started with mpMRI and was similar thereafter. In the third strategy, PSAD was calculated in case of a positive mpMRI result. The results confirmed that 4Kscore combined with mpMRI gave a better AUC = 0.82 than each method alone: 4Kscore (AUC = 0.70), mpMRI (AUC = 0.74). The best strategy seems to be an initial biopsy if the 4Kscore was >7.5%, followed by mpMRI and another biopsy for those with positive mpMRI (PIRADS ≥ 3) or 4Kscore >18%. This would avoid 34.2% of prostate biopsies while missing 2.7% of clinically significant PCa. However, this model is more expensive and requires external validation in a multicentre study, but it gives us an idea of how we can improve the selection of men for biopsy using biomarkers and mpMRI.

PHI, total PSA, PSAD and the ability of mpMRI to identify csPCa were compared in a group of 395 men [100]. In detecting csPCa for PSA, PSAD, Pi-RADS and PHI, the AUCs were as follows, respectively: 59.5, 64.9, 62.5 and 68.9 in patients undergoing biopsy, and for patients with a previous negative biopsy: 55.4, 69.3, 64.4 and 71.2. This indicates that PHI had comparable results to mpMRI and outperformed other indices.

Adding PHI to mpMRI leads to increased predictive accuracy of csPCa and a reduction of up to 50% in unnecessary biopsies (for men with PI-RADS 3–5 and PHI ≥ 30). Moreover, combination AUC outperforms PHI and mpMRI alone (AUC were 0.87, 0.73 and 0.83, respectively [101].

A study [102] performed prostate cancer diagnosis using a combination of Stockholm3 and mpMRI. Targeted biopsies or mpMRI were performed only in men at higher risk as assessed by S3M. When maintaining the number of detected FG cancers ≥2, there was a 42% saving of biopsies and a 46% reduction in FG1 detection. Using a combination of S3M and MRI TBx, the detection of GG 1 tumours and the number of biopsies needed were almost halved, with no reduction in sensitivity in detecting GG 2 cancers, compared with using SBx.

## 9. Discussion

PSA is a highly sensitive screening test. However, it lacks specificity, resulting in a high rate of unnecessary prostate biopsies. The liquid biopsy tests are more expensive than the commonly used PSA, but because of their better diagnostic value, they can be performed less frequently and avoid other more costly procedures such as biopsy or mpMRI.

It seems important to differentiate the tests in terms of their advantages and disadvantages and to demonstrate which biomarker may be most useful in a given clinical situation.

PHI, 4Kscore, SelectMDx and ExoDx offer better specificity than PSA and can help identify men with GS ≥ 7 PCa. MPS also outperforms PSA and each of its components in HG PCa detection, and its performance in men with suspicious PSA levels helps to validate the need for initial or repeat biopsy.

STHLM3 is also significantly superior to PSA and can detect HG PCa even in men with PSA levels ≥ 1.5 ng/mL. However, this test has not yet been validated on multiethnic groups, nor have tests comparing it with other liquid biopsy tests been developed.

In men at increased risk of PCa with a previous negative biopsy, additional information can be obtained with the Progensa-PCA3 urine test, MPS, ExoDx and the 4Kscore, PHI and STHLM3 serum tests or the tissue-based epigenetic test (ConfirmMDx).

PCA3 reduces prostate biopsy rates in men undergoing repeat biopsy, but there is still no consensus on the cut-off value.

As PCA3 increases with cancer aggressiveness, tests based on it—Progensa PCA3, MiPS and ExoDx—show the ability to distinguish between cancers with high and low Gleason scores, indicating high utility in therapeutic decision-making.

As ExoDx uses an algorithm independent of PSA and its derivatives, clinical factors (features) and standard of care (SOC), it is feasible (in the US) to perform at home. The patient takes a sample, hands it over to a courier and then discusses the result with the doctor via telehealth. This novelty (ExoDx Prostate At-Home Collection) seems particularly useful in times of coronavirus pandemics and for people living far from medical care.

PHI is significantly better than SelectMDx in diagnosing any PCa, while SelectMDx is significantly better than PHI in diagnosing csPCa.

The 4Kscore assesses the risk of detecting HG PCa if a biopsy is performed. It has been shown to have a better detection rate for HG PCa than the modified PCPTRC and SOC. In addition, 4Kscore can predict HG PCa even years in advance and assess the risk of distant metastasis, e.g., in genetically burdened men. It thus helps to non-invasively avoid prostate biopsy for men in whom it is not necessary and identifies men at higher risk for whom an early intervention is beneficial.

Hendriks [103] and colleagues undertook a comparison of the diagnostic values of two FDA-approved tests, PHI and PCA3, for primary and repeated biopsy. Unfortunately, after compiling all studies published before 2017, they were unable to draw clear conclusions due to the conflicting results of the articles analysed. Study [104] notes that although in a double-blind study of PCA3 vs. PHI, PCA3 is superior to PHI in cancer prediction accuracy, when considering only significant PCa, PHI remains the most accurate predictor. For this reason, the authors recommend using PHI instead of PCA3 in population-based screening.

In a study [54] on 531 men (PSA 3–15 ng/mL) who underwent an initial biopsy, 4Kscore and PHI had similar AUCs in predicting PCa (AUC = 0.69 and 0.74, respectively) and csPCa (0.72 vs. 0.71, respectively).

Russo et al. indicated in their systematic review [55] the high diagnostic accuracy of PHI and 4Kscore. Both tests were tested on multiethnic groups and showed high diagnostic value in them. Although both biomarkers provide similar diagnostic accuracy in the detection of general and high-grade PCa and reduce the number of unnecessary biopsies, it should be borne in mind that there are disturbing reports on PHI [80,81,82].

Furthermore, PHI should not be interpreted as absolute proof of the presence or absence of prostate cancer. Elevated PSA and PHI can be observed not only in patients with prostate cancer but also with benign diseases. PHI results should be interpreted taking into account clinical factors or family history, and individual clinical decisions should be made based on them.

Vedder et al. [105] added PCA3 and 4Kscore to the ERSCPC risk calculator and compared performance. They showed that 4Kscore was better than PCA3 in predicting PCA in men (with PSA ≥ 3.0 ng/mL) (AUC 0.78 and 0.62, respectively). However, when no PSA limit was set, PCA3 performed better than 4Kscore (AUC 0.63 vs. 0.56). When added to ERSPC, both biomarkers slightly improved the prediction of PCa, with no significant differences (in performance) between them.

Additionally, the previously mentioned study [60] confirmed that adding ERSPC to the 4Kscore improves diagnostic value. However, it is worth recalling that the 4Kscore is the most expensive of the tests compiled in our review.

In addition, it is important to remember that drugs such as 5-alpha-reductase inhibitors: finasteride, dutasteride and anti-androgen therapy can affect the levels of PSA and other biomarkers. Such medications should be discontinued for at least 6 months prior to the study. Samples for the test should be taken when the clinician is satisfied that the prostate tissue has recovered, normally no less than 6 months after the date of the last biopsy or any other prostate procedure. The impact of these procedures on the performance of the test has not yet been assessed.

## 10. Conclusions

Recently, molecular characterisation of PCa has become increasingly important, and a wide range of biomarker-based liquid biopsy tests are commercially available to assist urologists in clinical decision-making. The prostate cancer liquid biopsy biomarkers listed above have a high NPV and therefore help prevent unnecessary biopsies. As mentioned earlier, numerous publications [16,17,18] have not shown a correlation between PCA3 values and prostate cancer aggressiveness (Gleason score). Given this fact and reports of unexplained PCA3 well above the cut-off [15] without cancer on biopsy, it is reasonable to use newer, more sensitive and specific diagnostic tools to detect patients requiring prompt and radical treatment. For example, PCA3 in combination with other biomarkers such as TMPRSS2: ERG fusion [36] in Mi-Prostate Score [41,42,43,44,45,46] or ERG and SPDEF in ExoDx Prostate IntelliScore [47,48,61,62,63], where it shows better diagnostic and prognostic potential.

From a clinical point of view, it is critical to identify assays for the early detection of aggressive PCa subtype when it can still be treated effectively. Recent years have led to the development of totally non-invasive tests i.e., (ExoDx Prostate At-Home Collection) where first catch, nondigital rectal examination urine specimens appeared helpful in identifying aggressive (Gleason score 7–10) PCa in a racially diverse patient cohort. Similarly, the four-kallikrein panel showed effectiveness in identifying aggressive PCa in a multiethnic population.

It seems that in the near future, molecular biomarkers, clinical and histopathological features and diagnostic imaging will have to be used in a complementary rather than a competitive manner to ensure the best possible selection of patients for mpMRI and eventual biopsy.

## Abbreviations

AUC	Area under the curve
BMI	Body Mass Index
csPCa	Clinically significant prostate cancer
DCA	Decision curve analysis
DRE	Digital Rectal Examination
EAU	European Association of Urology
EPI	ExoDx Prostate IntelliScore
ERSPC RC	European Randomised study of Screening for Prostate Cancer risk calculator
fPSA	Free non-protein-bound PSA
GG	Grade Group
GS	Gleason score
HG	High grade
hK2	Human kallikrein 2
iPSA	Intact PSA
ISUP	International Society of Urological Pathology
LG	Low grade
lncRNA	Long non-coding RNA
MiPS	Michigan Prostate Score
mpMRI	Multiparametric Magnetic Resonance Imaging
MRI	Magnetic Resonance Imaging
NPV	Negative predictive value
NCCN	National Comprehensive Cancer Network
PCa	Prostate cancer
PCA3	Prostate Cancer Antigen 3
PCPT-RC	Prostate Cancer Prevention Trial Risk Calculator
PHI	Prostate Health Index
PHID	Prostate Health Index density
PI-RADS	Prostate Imaging-Reporting and Data System
PPV	Positive predictive value
PSA	Prostate-specific antigen
PSAD	Prostate-specific antigen density
QALY	Quality-adjusted life year
RP	Radical prostatectomy
SBx	Systematic Biopsy
SNPs	Single-nucleotide polymorphisms
SOC	Standard of care
STHLM3	Stockholm 3
S3M	Stockholm 3 Model
TBx	Targeted biopsy
TNM	Tumor-Node-Metastasis (Staging System)
tPSA	Total PSA
TRUS	Transrectal ultrasound
TRUS-Bx	Transrectal ultrasound (TRUS) guided biopsy
4Kscore	Four-kallikrein score
